# Case Report: Brugada syndrome uncovered by delivery in a 32-year-old patient

**DOI:** 10.3389/fcvm.2026.1902265

**Published:** 2026-09-09

**Authors:** Peng Liu, Ruilong Gao, Yaliu Yang, Zhengqin Zhai, Yifeng Zhou

**Affiliations:** Department of Cardiology, China-Japan Friendship Hospital, Beijing, China

**Keywords:** Brugada syndrome, cardiac channelopathy, peripartum period, sudden cardiac arrest, ventricular arrhythmias

## Abstract

We report a 32-year-old primigravida with no prior symptoms, normal baseline ECG, and no family history of sudden death who developed ventricular fibrillation (VF) shortly after uncomplicated vaginal delivery. She received epidural ropivacaine and sufentanil during labor. Post-resuscitation ECG demonstrated coved ST-segment elevation with T-wave inversion in V_1_–V_2_, consistent with spontaneous type 1 Brugada syndrome (BrS) pattern. Echocardiography excluded structural heart disease; coronary CT angiography excluded coronary artery disease. Laboratory investigations revealed borderline hypokalemia (3.5 mmol/L) and mild hypomagnesemia (0.75 mmol/L). Clinical whole-exome sequencing revealed no pathogenic or likely pathogenic variants in established arrhythmia-associated genes. The ECG normalized two days later. An implantable cardioverter-defibrillator was implanted. This case illustrates multifactorial unmasking of latent BrS—involving ropivacaine-mediated sodium channel blockade, postpartum autonomic instability, bradycardia, and electrolyte imbalance in a genotype-negative asymptomatic woman, highlighting the importance of recognizing concealed channelopathies in peripartum cardiac arrest.

## Introduction

Brugada syndrome (BrS) is an inherited cardiac channelopathy characterized by pathognomonic coved type ST-segment elevation and T-wave inversion in the right precordial leads, predisposing affected individuals to malignant ventricular arrhythmias or sudden cardiac death (SCD) (1). The prevalence of BrS varies markedly among sexes and ethnic groups, with men having 8–10-fold higher prevalence than women (2). Although loss-of-function variants in *SCN5A* represent the most established genetic substrate, pathogenic mutations are identified in only 20%–30% of clinically diagnosed cases (3). BrS may remain clinically silent until exposure to precipitating factors such as sodium-blocking agents, fever, autonomic fluctuations, and electrolyte disturbances. Pregnancy and the peripartum period represent a unique physiological state characterised by profound hormonal, haemodynamic, and autonomic changes that may destabilise latent arrhythmogenic substrates. We report a previously asymptomatic woman with normal baseline electrocardiogram (ECG) who developed ventricular fibrillation (VF) due to transient unmasking of BrS shortly after vaginal delivery. This case highlights the multifactorial interaction between epidural ropivacaine exposure, postpartum autonomic instability, and electrolyte imbalance in precipitating malignant ventricular arrhythmia.

## Case description

**Figure 1:**
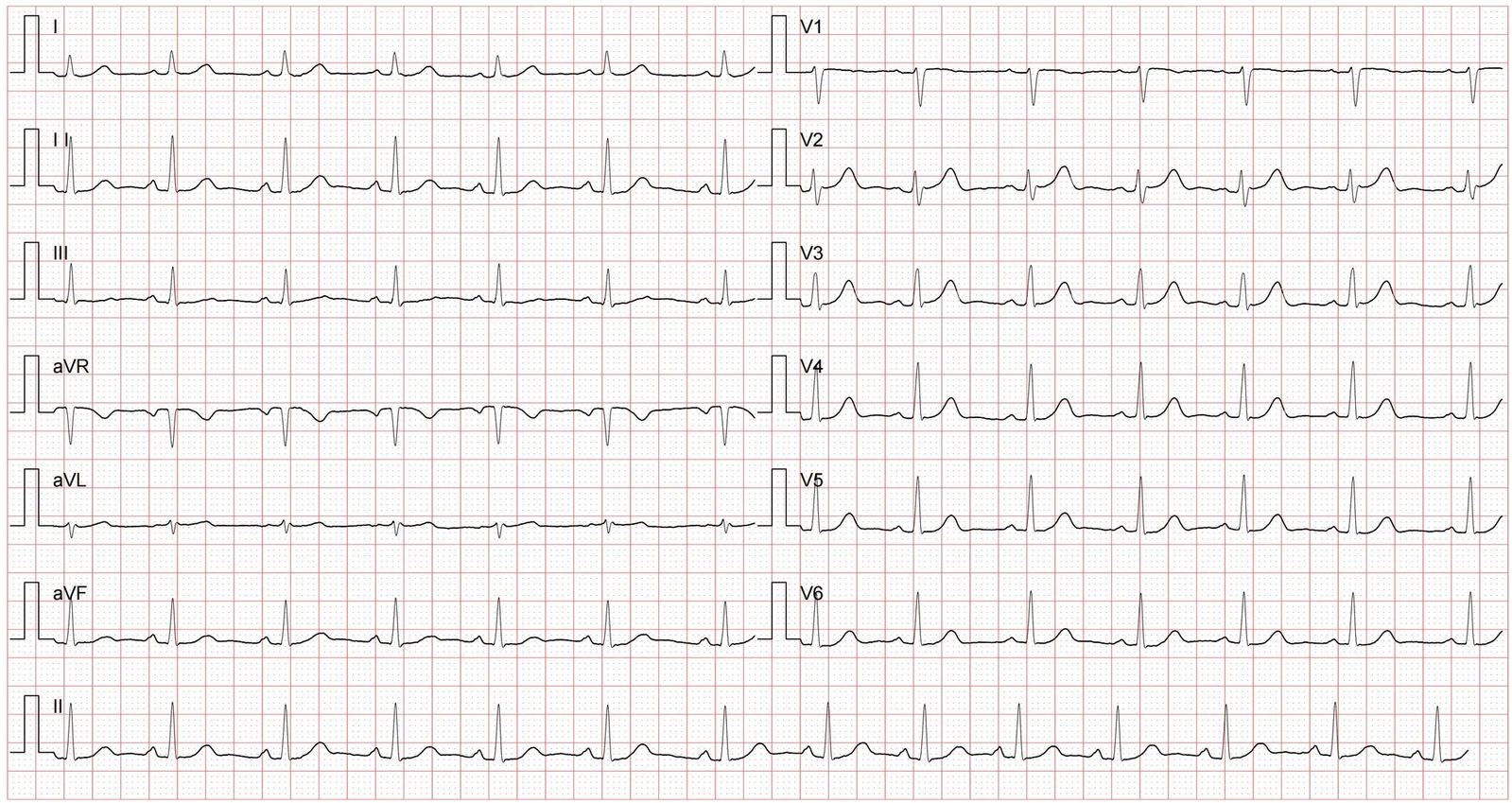
ECG on admission was within normal limits.

A 32-year-old primigravida woman at 38 weeks and 5 days of gestation was admitted for spontaneous labor. She underwent epidural analgesia with ropivacaine and sufentanil during a 3-hour labor followed by uncomplicated vaginal delivery. Her body temperature remained normal throughout labor with no fever recorded. She had no prior history of syncope, seizures, palpitations, or cardiac arrest, and no family history of SCD or inherited arrhythmia syndromes. Baseline ECG on admission was normal (Figure 1).

Following delivery, she complained of palpitations. Approximately 90 min postpartum, she suddenly developed seizures and loss of consciousness. ECG monitoring documented ventricular fibrillation. Return of spontaneous circulation was achieved after immediate defibrillation and cardiopulmonary resuscitation, after which she was transferred to the intensive care unit.

**Figure 2:**
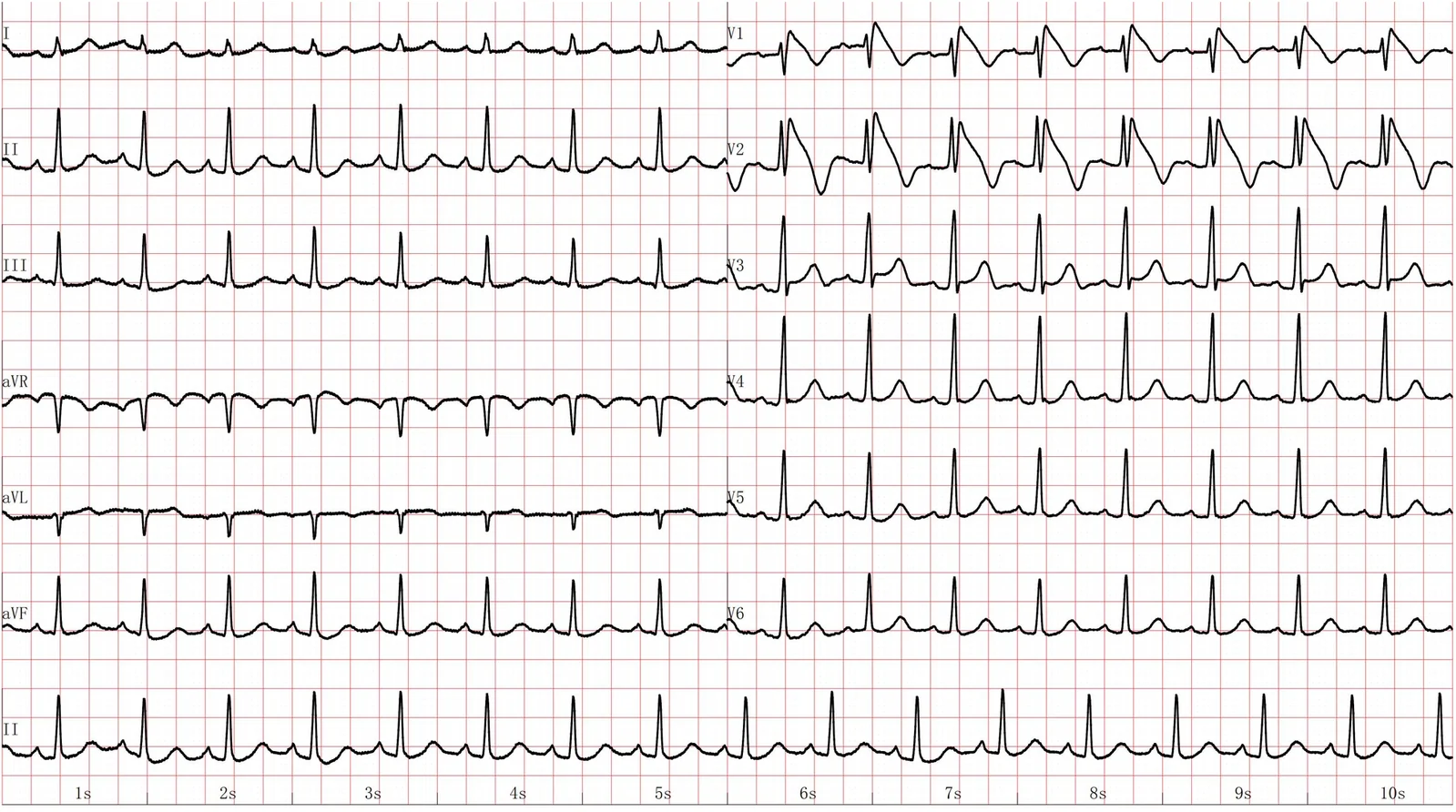
Coved ST-segment elevation with T-wave inversion in lead V1 and V2, suggestive of brugada syndrome type 1.

Post-resuscitation ECG demonstrated coved ST-segment elevation with T-wave inversion in leads V1 and V2, consistent with spontaneous type 1 Brugada ECG pattern (Figure 2). Transthoracic echocardiography showed no structural heart disease or evidence of pulmonary embolism. Laboratory investigations revealed borderline hypokalemia (serum potassium 3.5 mmol/L) and mild hypomagnesemia (0.75 mmol/L). Neurological evaluation was unremarkable.

**Figure 3:**
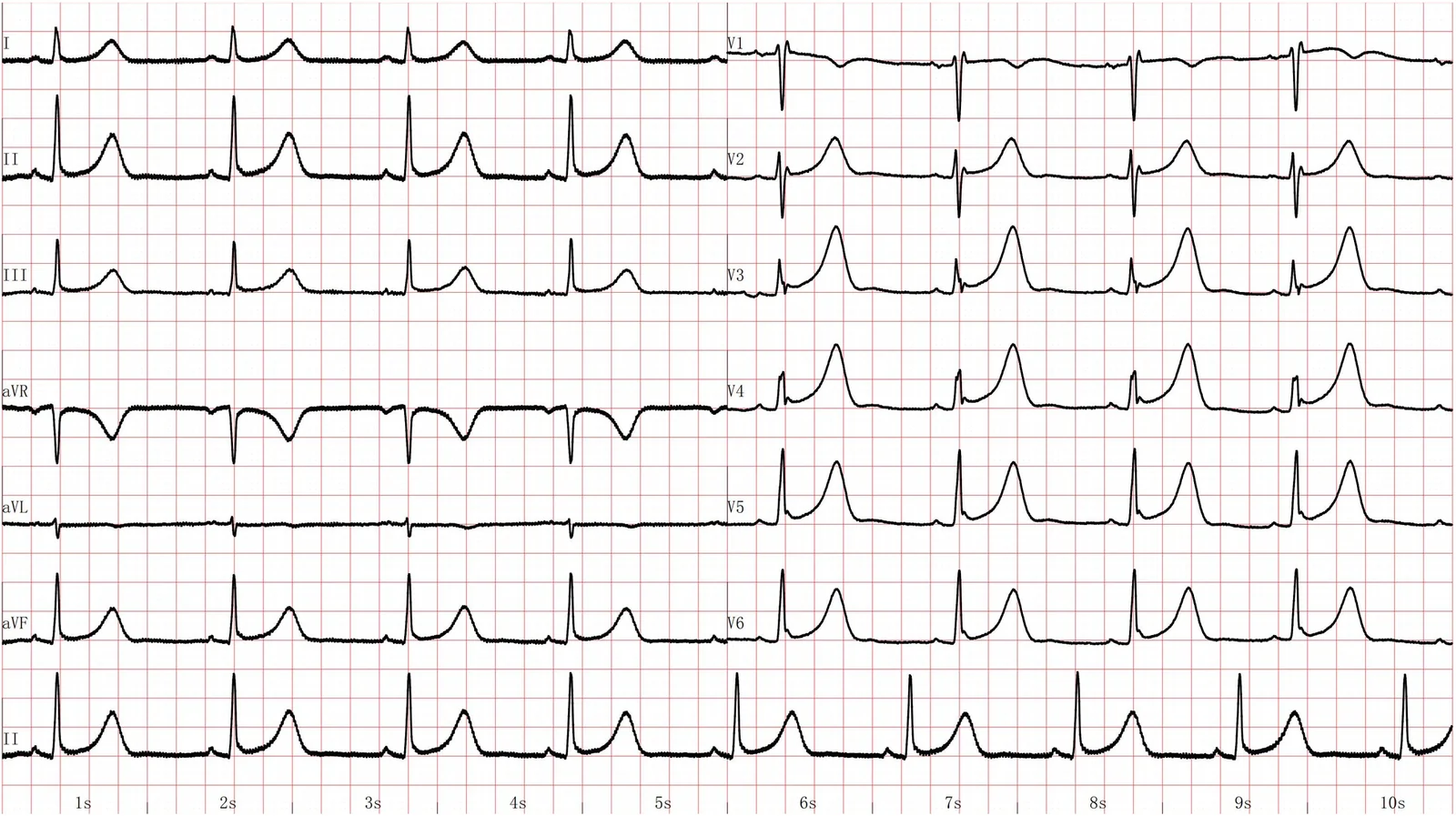
Sinus bradycardia of 51 beats per minute, prolonged QTc interval of 521 ms, incomplete right bundle branch block and slight ST-segment elevation in precordial leads.

The following morning, repeat ECG demonstrated sinus bradycardia (51 beats per minute), prolonged QTc interval (521 ms), incomplete right bundle branch block, and persistent mild ST-segment elevation in the right precordial leads (Figure 3). Intravenous magnesium sulfate was administered. Two days after the cardiac arrest, the ECG normalized completely.

Coronary CT angiography excluded coronary artery disease. Clinical whole-exome sequencing (WES) of peripheral blood DNA was performed (BGI Genomics, Shenzhen, China). No pathogenic or likely pathogenic variants were identified in *SCN5A* or any other established arrhythmia- or BrS-susceptibility gene, and no phenotype-related copy number variants or mitochondrial DNA variants were detected. Given the documented VF arrest and spontaneous type 1 Brugada ECG pattern, a single-chamber transvenous implantable cardioverter-defibrillator (ICD; INOGEN™ EL ICD VR, model D140, Boston Scientific, Marlborough, MA, USA) was implanted one week later for secondary prevention. The device was programmed with a ventricular fibrillation zone at ≥ 230 bpm. The patient was discharged two days after ICD implantation. During 18 months of follow-up, scheduled outpatient device interrogations revealed no ventricular arrhythmic episodes, no delivered antitachycardia pacing or shock therapies, and no arrhythmia electrograms were recorded.

## Discussion

This case illustrates malignant postpartum unmasking of previously silent Brugada syndrome in a young woman without prior symptoms, baseline ECG abnormalities, family history, or identifiable pathogenic mutations. The temporal relationship between labor, epidural anesthesia, and ventricular fibrillation strongly suggests a multifactorial reduction in arrhythmic threshold rather than a single isolated trigger.

The electrophysiological substrate of BrS arises from an imbalance between inward depolarising currents and outward repolarising currents within the right ventricular outflow tract epicardium, resulting in exaggerated transmural dispersion of repolarisation and susceptibility to phase 2 re-entry (4, 5). A retrospective study of 104 patients showed no increased risk of arrhythmic events during pregnancy in women with BrS (6). In the present case, several transient factors likely converged to destabilise a previously compensated substrate.

Ropivacaine, although generally considered less cardiotoxic than bupivacaine, exerts dose-dependent blockade of cardiac sodium channels. Systemic absorption during prolonged epidural infusion may reduce sodium current sufficiently to unmask latent BrS in susceptible individuals (7, 8). Previous reports have linked local anesthetics, particularly bupivacaine, to Brugada-type ECG changes and malignant ventricular arrhythmias (9, 10). Because re-exposure for diagnostic confirmation is neither feasible nor ethical, the association between ropivacaine and Brugada unmasking in this patient is necessarily inferential, based on the temporal sequence of events, the known pharmacology of the agent, and previously published reports. Although direct causality cannot be definitively established in this patient, ropivacaine exposure likely contributed to reduction of depolarisation reserve.

Autonomic fluctuation during labor and the immediate postpartum period probably represented an additional critical trigger. Repeated Valsalva manoeuvres during the second stage of labor may transiently enhance vagal activity, while abrupt postpartum haemodynamic redistribution can further destabilise cardiac electrophysiology. Increased vagal tone is known to accentuate Brugada ECG manifestations by augmenting outward currents and promoting loss of the epicardial action potential dome (11).

Reflex mechanisms warrant consideration in young, asymptomatic individuals with BrS. Arrhythmic syncope is rare in BrS, whereas reflex syncope occurs at least as often as in the general population, particularly during pregnancy and the peripartum period (12). Distinguishing the two is essential, as misclassification directly alters risk stratification and management. Increased vagal tone may be doubly relevant in this setting, both triggering reflex events and accentuating the Brugada phenotype (11). In this case, the index event was a monitored VF arrest, leaving no diagnostic ambiguity. If syncope occurs during long-term follow-up, device interrogation and, if necessary, head-up tilt testing should be performed to clarify its mechanism.

Borderline hypokalemia and mild hypomagnesemia may also have lowered the ventricular fibrillation threshold. Although electrolyte abnormalities were modest, even subtle disturbances may exacerbate repolarisation instability when superimposed on latent channelopathy (13).

Taken together, the clinical presentation likely reflected a “perfect storm” in which sodium channel blockade, postpartum autonomic instability, haemodynamic changes, bradycardia, and electrolyte imbalance collectively unmasked a concealed arrhythmogenic substrate.

BrS demonstrates marked sex disparity, with men exhibiting substantially higher prevalence and arrhythmic risk (14, 15). Female patients more commonly remain asymptomatic and less frequently exhibit spontaneous type 1 ECG patterns (15). In this patient, female sex may have increased the threshold for clinical manifestation, allowing the substrate to remain silent until exposure to multiple acute stressors.

Notably, whole-exome sequencing identified no pathogenic or likely pathogenic variants in *SCN5A* or other established BrS-susceptibility genes. However, genotype-negative BrS remains common, as pathogenic variants are identified in only a minority of clinically diagnosed patients (3, 16). Therefore, absence of detectable mutations does not exclude the diagnosis when characteristic ECG findings and clinical presentation are present.

Device selection is particularly relevant in patients with drug-induced or transiently unmasked type 1 BrS, who are typically young and rarely require pacing. A meta-analysis of randomized and propensity score-matched studies showed comparable efficacy and overall safety between the subcutaneous ICD (S-ICD) and the transvenous ICD (TV-ICD), with fewer lead-related complications for the S-ICD (17), and contemporary intermuscular implantation techniques have further improved S-ICD performance (18). However, the Brugada ECG may alter QRS/T-wave morphology and increase the risk of T-wave oversensing, so careful multi-vector screening is required before S-ICD implantation in this population. In this case, a single-chamber TV-ICD was chosen for several reasons: sinus bradycardia and transient QTc prolongation during the index hospitalization, which favored a system retaining pacing capability; and the longevity and lower cost of the transvenous device, an important consideration in a young patient facing decades of device therapy. Over 18 months of follow-up, no device-related complication, infection, or inappropriate therapy occurred. Furthermore, given the transient and multifactorial nature of the unmasking triggers in this genotype-negative patient, long-term device management remains open to reassessment. If no arrhythmic events are documented throughout the life of the first device, the option of forgoing generator replacement or even removing the device and its lead may be reconsidered at the time of battery depletion, weighing the uncertain risk of arrhythmia recurrence against the cumulative risks of lifelong device therapy. Regular surveillance and strict avoidance of precipitating factors would remain essential regardless of that decision.

This case has several limitations. First, a pharmacological challenge test with sodium channel blockers (e.g., flecainide) was not performed during the recovery period or after ECG normalization, which would have provided additional confirmatory evidence for the diagnosis of concealed Brugada syndrome. Second, an invasive electrophysiological study with programmed ventricular stimulation was not conducted, precluding risk stratification and assessment of arrhythmia inducibility. Third, although comprehensive genetic testing for *SCN5A* and other major arrhythmia-related genes was negative, the possibility of variants in currently unidentified or novel disease-associated genes cannot be excluded. In addition, whole-exome sequencing does not cover non-coding regions such as promoters and cannot exclude somatic mosaicism, structural variants, or a polygenic contribution to arrhythmia susceptibility. Finally, serum ropivacaine concentrations were not measured during the perioperative period, preventing definitive establishment of a dose–response or causal relationship between local anesthetic exposure and Brugada unmasking.

## Conclusion

This case demonstrates life-threatening ventricular fibrillation in the context of multifactorial unmasking of Brugada syndrome following vaginal delivery in a previously asymptomatic woman with normal baseline ECG and negative genetic testing. The convergence of ropivacaine-mediated sodium channel blockade, postpartum autonomic fluctuation, bradycardia, haemodynamic changes, and electrolyte imbalance likely destabilised a latent arrhythmogenic substrate. The case highlights the importance of recognising concealed channelopathies in peripartum cardiac arrest and underscores the need for multidisciplinary awareness during obstetric management.

## Data Availability

The datasets presented in this study can be found in online repositories. The names of the repository/repositories and accession number(s) can be found in the article/Supplementary Material.
